# Renal Infarcts—A Perplexing Case in the Middle of the COVID-19 Pandemic

**DOI:** 10.3389/fped.2021.669453

**Published:** 2021-05-14

**Authors:** Brett Plouffe, Tamara Van Hooren, Michelle Barton, Nancy Nashid, Erkan Demirkaya, Kambiz Norozi, Irina Rachinsky, Johan Delport, Michael Knauer, Soumitra Tole, Guido Filler

**Affiliations:** ^1^Department of Pediatrics, Schulich School of Medicine and Dentistry, University of Western Ontario, London, ON, Canada; ^2^Children's Health Research Institute, Lawson Health Research Institute, University of Western Ontario, London, ON, Canada; ^3^Department of Pediatric Cardiology, Medical School Hannover, Hannover, Germany; ^4^Department of Imaging, Schulich School of Medicine and Dentistry, University of Western Ontario, London, ON, Canada; ^5^Departments of Medicine Pathology and Laboratory Medicine, University of Western Ontario, London, ON, Canada; ^6^Lilibeth Caberto Kidney Clinical Research Unit, Lawson Health Research Institute, University of Western Ontario, London, ON, Canada

**Keywords:** renal infarct, multisystem inflammatory syndrome in children, pediatric inflammatory multisystem syndrome, COVID-19, SARS-CoV-2, merged SPECT/CT

## Abstract

Renal infarction is a rare finding in children. Associations between SARS-CoV-2 infections and thromboembolic events including renal infarcts have been described in adults. Although a similar association in children has not yet been described with this pandemic, the pediatric literature is still evolving with the recognition of new manifestations including the post-infectious Multisystem Inflammatory Syndrome in Children (MIS-C). We report the rare event of multiple renal infarcts in a 6-year-old boy manifesting several features of MIS-C 9 weeks following a self-limiting febrile illness characteristic of COVID-19. An underlying Factor V Leiden mutation was identified in this child but felt to be insufficient on its own to explain his clinical presentation. As SARS-CoV-2 testing was delayed, the failure to identify viral RNA or antibodies may not exclude the virus' potential role in precipitating the infarct in this host. Given that renal infarcts have been described in adult patients with COVID-19, reporting this perplexing case where SARS-CoV-2 may have played a role, may help identify this potential complication.

## Background

Acute renal infarcts in children are rare and relate to cardiac conditions (atrial fibrillations/flutter, or valve vegetations), thrombi from atheroma, renal artery dissection, fibromuscular dysplasia, hypercoagulability, renal trauma, and rare systemic or infectious diseases (1). They are usually embolic and observe segmental morphology as a function of renal anatomy, resulting in a wedge-shaped region of decreased enhancement in MRI or CT imaging (2). In a systematic review of the adult literature, 45% of renal infarction was caused by cardiac reasons or aortic embolism, 16% arterial injury, 9% prothrombotic factors in 9 and 21% miscellaneous or idiopathic (3). Recently, COVID-19 infections have been added to the list of causes for renal infarcts (4–7).

## Case Report

A previously healthy 6-year-old Middle Eastern boy with a negative history of consanguinity, antenatal anomalies of the urinary tract or urinary tract infections, presented with a one-day history of severe abdominal/right-sided flank pain, vomiting and fever (40.5°C). Parents denied trauma, dysuria, Kawasaki-like features, or recent ill contacts. Nine weeks earlier, he had a self-limiting 7-day-febrile illness with pharyngitis, dry cough and myalgia. No SARS-CoV-2 testing was performed at that time.

### Labs

The initial white blood cell count was 16.0^*^10^∧^9/L (normal ^*^5.0–12.0) without a left shift and platelets peaked at 664^*^10^∧^9/L on day 10. Transaminases, albumin, creatine kinase, lactase dehydrogenase and troponin were normal. Serum creatinine was not elevated and the improved Schwartz formula eGFR was normal. C-reactive protein peaked at 340.0 mg/L (normal <5.0) and ferritin was 285 ug/L. Fibrin D-dimer was 2,843 ug/L (normal <499). Lupus serology, anti-thrombin, protein C and S assays, p and c-ANCA, rheumatoid factor and lipid profile were all normal. Interferon-gamma release assay and workup for antiphospholipid antibody syndrome and hyperhomocystinemia were negative. However, the patient tested positive for a heterozygous factor V Leiden mutation (c.1691G>A) (FVL). Urinalysis showed transient microhematuria, and urine cultures remained sterile. SARS-CoV-2 PCR was negative for the patient as were anti-SARS-CoV-2 total, IgG and IgA assays for the patient and his family.

### Imaging

**Figure 1:**
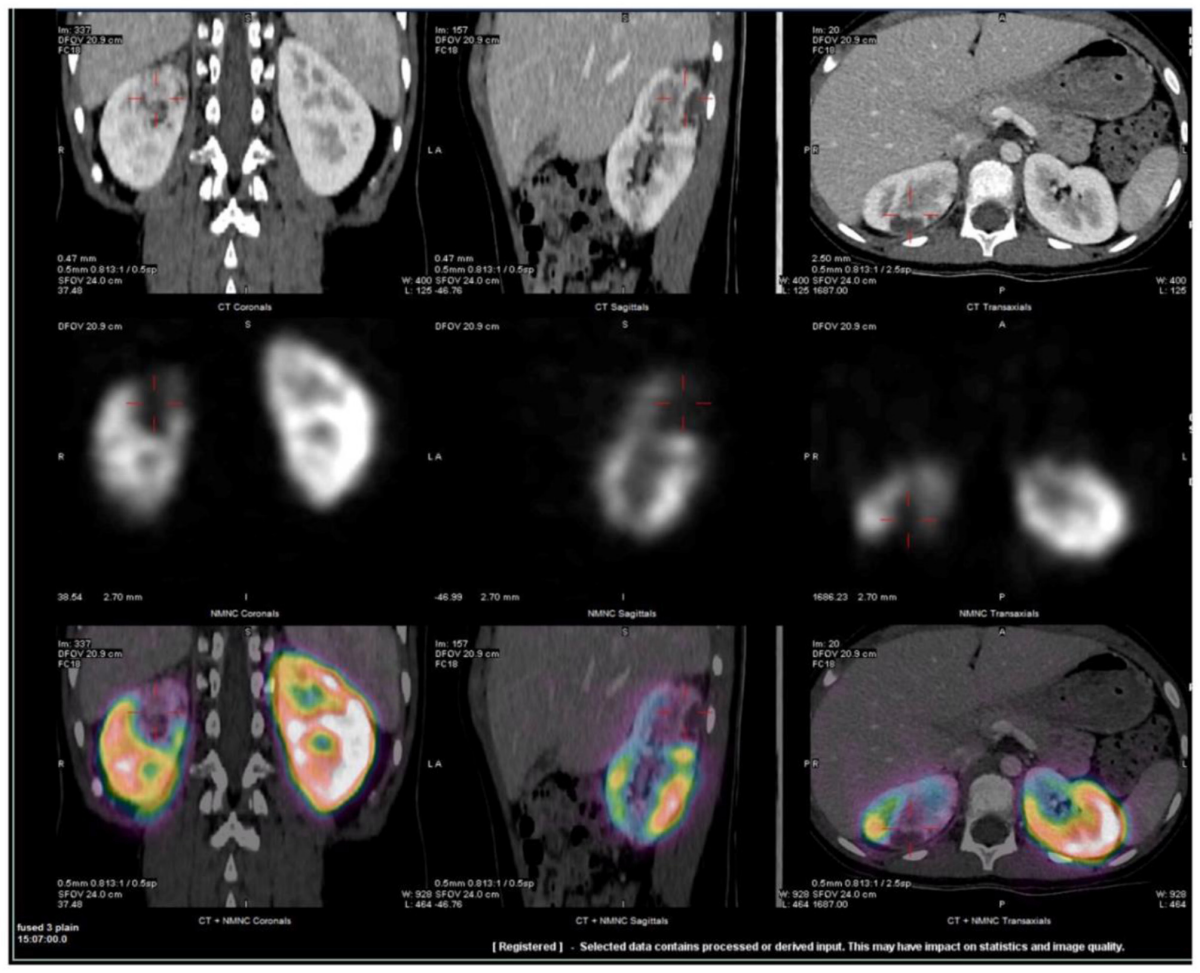
Representative fused SPECT/CT images of Tc99m-DMSA scan. CT scan was obtained with IV contrast. Images demonstrate wedge-shaped cortical defect involving the superior pole of the right kidney compatible with infarct. Two additional, smaller defects were also visualized in the lower and mid poles (not shown). Upper row: CT images, Middle row: Tc99m –DMSA scan and Lower row: fused SPECT/CT images in coronal, sagittal and axial projections.

Chest X-ray and echocardiogram were normal. The abdominal ultrasound showed no evidence for appendicitis or renal lesions. His abdominal CT demonstrated abnormal peripheral hypo-enhancing areas in the upper pole of the right kidney (Figure 1). An MRI pre- and post-gadolinium also demonstrated multiple wedge-shaped areas of decreased enhancement in the right upper pole. Both MRI and CT raised concerns for systemic autoinflammatory process given the finding of bilateral small volume pleural effusions, ascites, appendiceal inflammation and distended gall bladder. No vessel abnormalities were detected, especially no aneurysms. Tc 99-DMSA renal scan demonstrated a normal cortical uptake on the left kidney whereas the right kidney demonstrated wedge-shaped defects in the posterior aspect of the upper pole (Figure 1).

Empiric ceftriaxone was discontinued as all cultures were sterile. Fever and pain persisted for 7 days. The final diagnosis was idiopathic acute renal infarction. On follow up, the patient remained asymptomatic and was kept on 81 mg of Aspirin for 6 months.

## Discussion

This 6-year-old boy suffered acute renal infarctions without a clear identifiable etiology. Abdominal pain, flank pain, nausea, vomiting, fever as well as the course of the C-reactive protein and the D-dimer are typical characteristics of acute renal infarction (1). The only disposing factor was a heterozygous state for factor V Leiden c.1691G>A mutation, and while this condition has been associated with neonatal renal vein thrombosis (8), thrombotic events on the arterial side are very rare outside of renal transplantation. Thrombophilia in idiopathic renal infarction has been reported primarily in older adults, and related to antiphospholipid antibody syndrome, hyperhomocystenemia, or combined thrombophilias (1, 9–11). While there have been conflicting studies, particularly in children, two large meta-analyses have shown no association between FVL and arterial thrombosis (9, 12). While it is possible that FVL may have interacted with other genetic and environmental factors, it is our opinion that this mutation on its own is unlikely to have been the reason for the renal infarcts.

In the context of the COVID-19 pandemic, given this patient's multi-visceral inflammation and antecedent illness consistent with COVID-19, the multi-system inflammatory syndrome in children (MIS-C) was considered. Several countries affected by the coronavirus disease (COVID-19) pandemic recently reported cases of children that were hospitalized in intensive care due to a rare MIS-C, also known as pediatric inflammatory multisystem syndrome (PIMS). The presenting signs and symptoms are a mix of the ones for Kawasaki disease and toxic shock syndrome and are characterized, among others, by fever, abdominal pain and cardiac involvement (13, 14). Kidney involvement has been described, (15) albeit in most cases as acute kidney injury (16). On 1 May 2020, the Royal College of Paediatrics and Child Health published a guidance document on the clinical management of children presenting with PIMS-TS and proposed the following case definition (17):

- A child presenting with persistent fever, inflammation (neutrophilia, elevated CRP and lymphopaenia) and evidence of single or multi-organ dysfunction (shock, cardiac, respiratory, renal, gastrointestinal orneurological disorder) with other additional clinical, laboratory or imaging and ECG features. Children fulfilling full or partial criteria for Kawasaki disease may be included.
- Exclusion of any other microbial cause, including bacterial sepsis, staphylococcal or streptococcal shocksyndromes, infections associated with myocarditis such as enterovirus.
- Proof of recent SARS-CoV 2 infection (positive pCR or antibody test) or epidemiological link.
  As such, our patient would meet the definition. However, testing for SARS-CoV-2 antibodies was negative 12 weeks post-COVID-19-like illness. Interpretation of these results are challenging given that the serology results may be falsely negative or become negative because of waning immunity over time (18, 19). Furthermore, laboratory evidence of recent or past SARS-CoV-2 infection may be lacking in children with MIS-C (15). In view of his antecedent illness and no other identified risk factor for his renal infarcts, we wonder if this perplexing case may be related to a past SARS-CoV-2 infection, as it has been described in adults (5, 7). MIS-C and other COVID-19-related events may be underreported given the limitation of diagnostic tests to confirm past COVID-19 infections in children. Renal infarction is such a rare event in otherwise healthy children as well as those with FVL mutations, that when associated with a febrile multisystem illness during the pandemic there should be a high index of suspicion about MIS-C triggering thrombotic events, even without the confirmed presence of SARS-CoV-2 antibodies. It is our hope that reporting this case increases the body of evidence about this rare pediatric condition.

## Consent to Participate

The authors declare that they have obtained consent to participate from the caregiver of the study.

## Author Contributions

BP, NN, KN, ED, MK, JD, IR, and ST provided major intellectual input into the design of the study, helped with the interpretation of the results, carefully edited and revised the various versions of the manuscript and approved the final manuscript. TV worked with the senior author on the analysis and collation of all differential diagnoses, helped with the drafts, provided vital intellectual input in the various versions and approved the final manuscript. MB co-conceptualized the design of the study, was instrumental in initiating critical investigations, helped with the interpretation of the results, and played a critical role in redrafting the manuscript with substantial edits of various versions of the manuscript and approved the final manuscript. IR fused the imaging and generated Figure 1. GF conceived this project, wrote the drafts, collated the results, made multiple edits, collated all changes, added intellectual content and approved the final version. All authors approved the final manuscript as submitted and agree to be accountable for all aspects of the work.

## Conflict of Interest

The authors declare that the research was conducted in the absence of any commercial or financial relationships that could be construed as a potential conflict of interest.
